# Arterial stiffness and hypertension

**DOI:** 10.1186/s40885-023-00258-1

**Published:** 2023-12-01

**Authors:** Hack-Lyoung Kim

**Affiliations:** Division of Cardiology, Department of Internal Medicine, Boramae Medical Center, Seoul National University College of Medicine, 5 Boramae-Ro, Dongjak-Gu, Seoul, 07061 Republic of Korea

**Keywords:** Arterial damage, Arterial stiffness, Cardiovascular risk, Hypertension, Target organ damage

## Abstract

Arterial stiffness and hypertension are closely related in pathophysiology. Chronic high blood pressure (BP) can lead to arterial wall damage by mechanical stress, endothelial dysfunction, increased inflammation, oxidative stress, and renin–angiotensin–aldosterone system (RAAS) activation. Hypertension also increases collagen fiber production and accelerates elastin fiber degradation. Stiffened arteries struggle with BP changes, raising systolic BP and pulse pressure. The resulting increased systolic pressure further hardens arteries, creating a harmful cycle of inflammation and calcification. Arterial stiffness data can predict target organ damage and future cardiovascular events in hypertensive patients. Thus, early detection of arterial stiffness aids in initiating preventive measures and treatment plans to protect against progression of vascular damage. While various methods exist for measuring arterial stiffness, pulse wave velocity is a non-invasive, simple measurement method that maximizes effectiveness. Healthy lifestyle changes, RAAS blockers, and statins are known to reduce arterial stiffness. Further research is needed to ascertain if improving arterial stiffness will enhance prognosis in hypertensive patients.

**Graphical Abstract:**

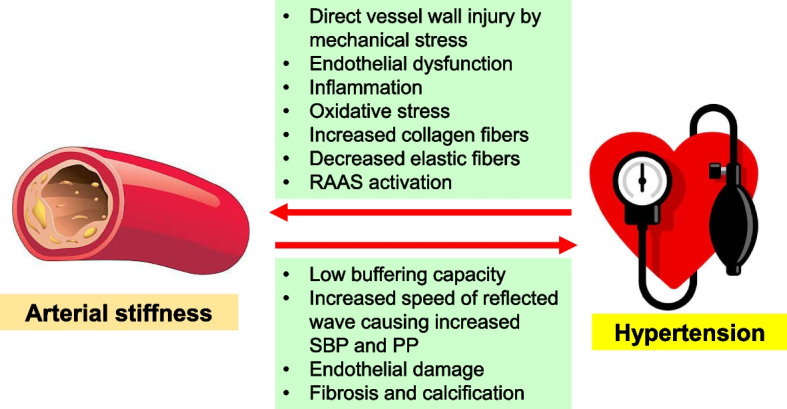

## Introduction

Hypertension, commonly known as high blood pressure (BP), is a prevalent medical condition where the force of the blood against the artery walls is consistently too high. This chronic condition is a significant global health burden because it is associated with an increased risk of cardiovascular and cerebrovascular diseases, which are among the leading causes of death worldwide [1, 2].

With age or due to prolonged exposure to cardiovascular disease risk factors, such as high BP, hyperglycemia, dyslipidemia, smoking, and chronic inflammation, the arterial wall tends to stiffen [3, 4]. As arterial stiffness escalates, systolic pressure increases and diastolic pressure decreases because of the premature merging of the fast-reflected wave with the forward wave [5]. The increased systolic pressure triggers left ventricular hypertrophy (LVH), while the diminished diastolic pressure impairs coronary blood flow. Additionally, an increase in arterial stiffness contributes to an elevation in pulse pressure (PP), which can potentially damage the blood–brain barrier [6]. Furthermore, in the blood vessel wall with increased arterial stiffness, more elastic fibers are destroyed, and the pulsatile pressure from the heart is not properly buffered and transmitted to the target organ, causing organ damage [7]. Arterial stiffness and poor cardiovascular prognosis often share common risk factors, reinforcing the correlation between them. This increased in arterial stiffness, driven by these mechanisms, is associated with a poorer cardiovascular prognosis [8, 9]. Hence, gathering data on arterial stiffness is critical in predicting a patient’s cardiovascular prognosis.

Arterial stiffness and hypertension are intricately intertwined, each significantly influencing the other. By elucidating the relationship between arterial stiffness and hypertension, we can gain valuable insights that can be applied to the diagnosis, treatment, and prognosis of patients with hypertension. In this review, I will explore the complex interplay between arterial stiffness and hypertension, and discuss the clinical implications derived from this relationship.

### Pathophysiology of arterial stiffening

Arterial stiffness refers to the reduced ability of an artery to expand and contract in response to pressure changes. With age and certain diseases, there are changes in the structure of the arterial wall. The arterial wall is made up of three layers: the intima (inner layer), the media (middle layer), and the adventitia (outer layer). The media is primarily made up of smooth muscle cells and elastin. Over time, there is a reduction in the elastin content and an increase in collagen, leading to stiffening of the arteries. In addition, there can be calcification within the arterial wall, contributing to increased stiffness [4]. The endothelium, the innermost lining of blood vessels, plays a key role in vascular tone by releasing substances like nitric oxide (NO), which causes vasodilation. With age and disease, there can be endothelial dysfunction, leading to decreased NO production and increased production of vasoconstrictive substances, contributing to arterial stiffness [10]. Chronic inflammation and oxidative stress are thought to play a significant role in the pathophysiology of arterial stiffness [11, 12]. Inflammatory and oxidative stress markers are often elevated in conditions associated with increased arterial stiffness, like hypertension and diabetes [13]. Increased arterial stiffness results in higher systolic BP and PP, leading to LVH and further cardiovascular complications [6].

### How to measure arterial stiffness

There are numerous methodologies for evaluating arterial stiffness, among which pulse wave velocity (PWV) is the most widely accepted and validated. PWV quantifies the rate at which the arterial pulse travels through the arterial system—a faster pulse wave indicates increased arterial stiffness. The most commonly utilized PWVs are carotid-femoral PWV (cfPWV) and brachial-ankle PWV (baPWV). Initially developed and primarily used in the West, cfPWV is considered the gold standard for noninvasive arterial stiffness measurement, as it focuses on the elastic artery portion between the carotid and femoral arteries [14]. A wealth of clinical data supports its use. In contrast, baPWV was developed later in Japan and is mainly used by Asians. Since baPWV also encompasses the muscular arteries between the upper arm and ankle, it has been criticized for not exclusively reflecting the elastic components of arterial stiffness. Despite this, numerous studies have confirmed that baPWV correlates well with cfPWV values and aligns well with invasively measured results [15]. Importantly, the prognostic value of baPWV has been substantiated in many clinical studies [9, 16–27]. Further, it’s reported that baPWV may better represent the total resistance value than cfPWV, with regard to the left ventricle’s afterload [28]. While cfPWV may cause patient discomfort when locating the femoral or carotid artery and necessitates skilled examiners, baPWV is simple and convenient, requiring only a BP cuffs wrapped around the limbs. Thus, for mass screenings involving large numbers of individuals, baPWV has distinct advantages [6]. In general, it is suggested that a cfPWV ≥ 10 m/s is considered a high-risk finding [29–32]. For baPWV, values < 14 m/s are considered low risk, ≥ 14 and < 18 m/s as moderate risk, and ≥ 18 m/s as high risk [29, 31, 32]. The strengths, limitations, and reference values of cfPWV and baPWV are compared in Table 1.

**Table 1:** The strengths, limitations, and reference values of cfPWV and baPWV

	**cfPWV**	**baPWV**
Strength	Regarded as the gold standard among non-invasive methods for measuring arterial stiffness	Simpler and quicker to measure, making it suitable for mass screening
	Supported by extensive clinical data and is the most validated measure	Offers insights into both central and peripheral arterial stiffness, providing a comprehensive assessment
	Provides an assessment of central aortic stiffness	The equipment for baPWV is more affordable and readily available in many clinical settings
	Minimally influenced by lower limb arterial disease	More reproducible with reduced variability due to its straightforward operator technique
Limitation	Can be uncomfortable for subjects during measurement	Reflects a mixed assessment of both central and peripheral arterial stiffness
	Demands specialized training and is more time-consuming to perform	Susceptible to alterations from conditions affecting peripheral arteries, particularly lower limb diseases
	Less suitable for mass screening	May not be universally recognized or preferred for some research or clinical contexts
	Does not provide insights into peripheral arterial stiffness	Lacks standardized normal values that are universally accepted
	Might overestimate values in individuals with short stature	Predominantly utilized in Asian countries
Reference value	Abnormal ≥ 10 m/s	Normal < 14 m/s Borderline ≥ 14 and < 18 m/s Abnormal ≥ 18 m/s

*cfPWV* carotid-femoral pulse wave velocity, *baPWV* brachial-ankle pulse wave velocity

The augmentation index (AIx) quantifies the extent to which the reflected pulse wave enhances the primary forwarding pulse wave [33]. Influenced by both the pulse wave’s speed, which is impacted by arterial stiffness, and the timing of the wave reflection, AIx is often measured using pulse wave analysis of the radial artery waveform [34]. This waveform is subsequently employed to generate a central (aortic) pressure waveform.

PP, defined as the difference between systolic and diastolic BP, serves as a rudimentary surrogate marker of arterial stiffness [35]. Both brachial PP and central aortic PP can act as arterial stiffness indicators. However, PP can be influenced by various factors, and thus, it may not be as accurate as other measures, like PWV. There are invasive and non-invasive techniques to measure central arterial PP. Due to the costs, invasiveness, and ethical considerations, invasive methods are primarily reserved for patients undergoing invasive coronary angiography [36, 37]. Recently, a widely used non-invasive approach infers the aortic pressure by obtaining the radial artery’s waveform, which is known for its relative high accuracy [38].

Another less common method of evaluating arterial stiffness involves directly measuring changes in arterial diameter in response to BP variations. While not frequently used in clinical practice, this method may be utilized in research settings. Ultrasound techniques, computed tomography, or magnetic resonance imaging can capture these changes by measuring the maximal (systolic) and minimal (diastolic) arterial diameters [39].

### Effect of hypertension on arterial stiffness

#### Arterial damage

Chronic hypertension can lead to arterial damage. High BP exerts additional strain on the walls of blood vessels. This augmented mechanical stress can directly damage the endothelium, the inner lining of the blood vessels, and stimulate vascular remodeling, resulting in thicker, stiffer arteries [40]. Hypertension can also impair endothelial function by potentially reducing the production of NO, a molecule vital for maintaining vascular health through its ability to relax blood vessels and inhibit inflammatory processes. This endothelial dysfunction is a key step in the development of atherosclerosis [41]. Moreover, hypertension is associated with increased inflammation and oxidative stress, both of which can further damage the blood vessels. Inflammatory cells can infiltrate the vascular wall, promoting the formation of atherosclerotic plaques, while reactive oxygen species can degrade NO, impairing its vasodilatory function [42]. Hypertension can stimulate the activation of the renin–angiotensin–aldosterone system (RAAS), a hormone system responsible for regulating BP and fluid balance. Excessive activation of this system can result in vasoconstriction, retention of salt and water, and additional vascular damage [43].

#### Collagen and elastin changes

Hypertension can cause significant alterations to the collagen and elastin composition within arterial walls, thereby contributing to vascular stiffness and remodeling. Collagen and elastin are two key structural proteins found in the walls of arteries. While elastin provides elasticity, allowing the arterial wall to stretch and recoil with each heartbeat, collagen provides strength and stability. Chronic high BP can stimulate the production of collagen in the arterial wall [40]. The excessive collagen can replace the more elastic fibers, making the arteries more rigid and less able to expand and recoil with blood flow. This can increase the systolic BP, and contribute to a vicious cycle of worsening hypertension and arterial stiffness. Additionally, hypertension can also accelerate the degradation of elastin fibers [44]. Loss of elastin reduces the elasticity of the arterial wall, contributing to arterial stiffness. Hypertension can also increase the activity of matrix metalloproteinases (MMPs), a family of enzymes that break down extracellular matrix proteins, including collagen and elastin [45]. While some degradation of extracellular matrix proteins is normal, excessive MMP activity can disrupt the balance between collagen and elastin, contributing to arterial remodeling and stiffness.

### Effect of arterial stiffening on BP

The relationship between arterial stiffening and BP is bidirectional. As arterial stiffness increases, the capacity of the artery to expand and contract in response to fluctuations in BP decreases. Over time, this diminished elasticity can lead to an increase in systolic BP and PP. This is because arteries with greater stiffness are less capable of expanding to accommodate the surge of blood ejected from the heart during each heartbeat [46]. Moreover, in a stiffened artery, the pulse wave travels at a higher speed. As a result, the reflected wave returns to the heart more quickly, coinciding with the systolic phase (the heart’s contraction phase). This phenomenon increases systolic BP, decreases diastolic BP, and widens PP [6]. An increase in systolic BP can, in turn, further contribute to arterial stiffening. This happens through damage to the endothelial cells lining the artery walls, which triggers inflammation, fibrosis, and calcification [40]. This perpetuates a vicious cycle of arterial stiffening and high BP.

**Fig. 1:**
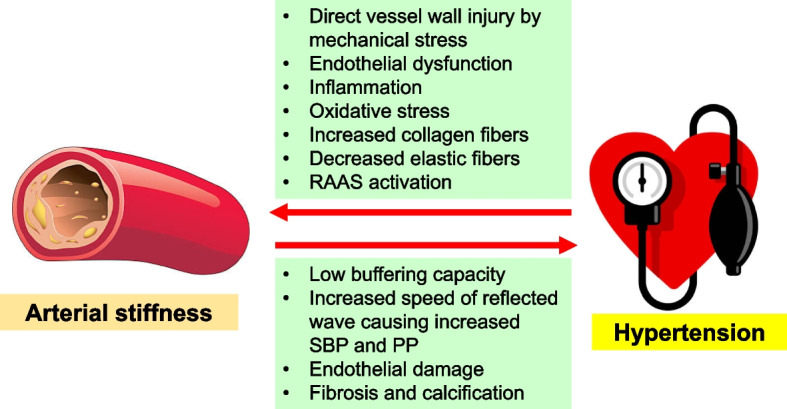
Pathophysiological correlation between arterial stiffness and hypertension. RAAS, renin–angiotensin–aldosterone system; SBP, systolic blood pressure; PP, pulse pressure

Figure 1 illustrates the pathophysiological correlation between arterial stiffness and hypertension.

### Implications of arterial stiffness in hypertensive patients

#### The role of arterial stiffness in predicting the future development of hypertension

Numerous studies suggest that arterial stiffness predicts future hypertension. Kaess et al. [47] discovered over a 7.8-year median follow-up that among 1,759 normotensive adults, increased arterial stiffness indicators like forward wave amplitude, AIx, and cfPWV predicted a higher hypertension risk. Another study involving 6,992 normotensive men found a 15% greater hypertension risk associated with carotid artery stiffness, regardless of baseline BP and other risk factors [48]. In a 4-year follow-up of 2,496 normotensive patients, those in the third tertile for baPWV were 3.5 times more likely to develop hypertension than those in the first tertile [49]. A study on 2,512 patients found a significant association between aortic elasticity and new hypertension cases over four years [50]. Finally, Najjar et al. [51] found that a 1 m/s PWV increase corresponded to a 10% rise in hypertension incidence among 449 normotensive individuals over 4.9 years.

These findings suggest the potential of arterial stiffness measures in early identification and prevention strategies for hypertension. However, further research is needed to determine how these measures can be best incorporated into routine clinical practice and public health efforts.

#### The role of arterial stiffness in the prediction of target organ damage of hypertensive patients

Arterial stiffness plays a significant role in the development and detection of target organ damage in hypertensive patients. Target organs in hypertension include the heart, kidneys, brain, and peripheral arteries [52]. Damage to these organs can lead to conditions such as heart disease, kidney failure, stroke, and peripheral artery disease [53]. Arterial stiffness can increase afterload, or the load against which the heart has to contract to eject blood. This can lead to LVH [6, 54], which is a thickening of the wall of the heart’s main pumping chamber. LVH is a common form of target organ damage in hypertension. Arterial stiffness can impair coronary perfusion, or the flow of blood to the heart muscle itself. This can lead to ischemic heart disease [5]. High arterial stiffness may cause high PP, which is a risk factor for cerebrovascular disease such as stroke [6]. The kidneys, as highly vascular organs, are also susceptible to damage from high BP and increased arterial stiffness, leading to kidney failure [55]. Increased arterial stiffness can also lead to peripheral artery disease, which is when the arteries supplying blood to the legs become narrowed or blocked [56]. In terms of detection, arterial stiffness can be measured using non-invasive techniques such as PWV, which is the speed at which the arterial pulse propagates through the circulatory system. An increased PWV is indicative of higher arterial stiffness. On these back grounds, some guidelines recommend PWV measurement as an optional test for early organ damage evaluation and risk prediction in hypertensive patients [30, 32, 57]. This makes it an important parameter to monitor in hypertensive patients for early detection and prevention of target organ damage.

**Fig. 2:**
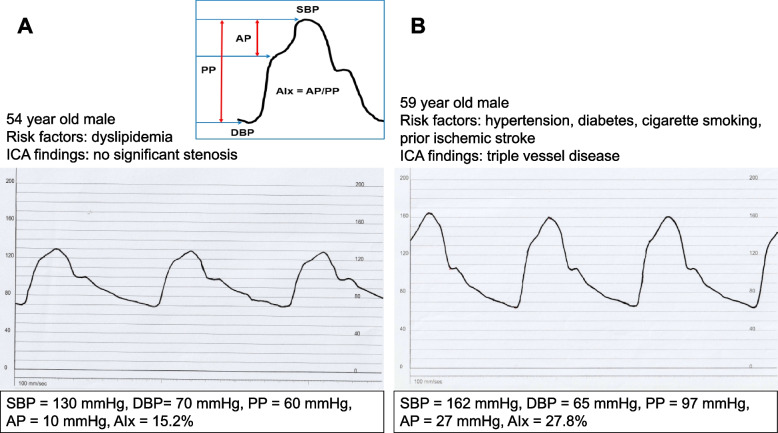
Invasively measured central aortic pressure waveforms and associated hemodynamic parameters in low-to-intermediate-risk (**A**) and high-risk (**B**) individuals. SBP, systolic blood pressure; AP, augmentation pressure; PP, pulse pressure; AIx, augmentation index; DBP, diastolic blood pressure; ICA, invasive coronary angiography

Figure 2 compares representative waveforms and hemodynamic parameters of invasively measured central aortic pressure curves between low-to-intermediate-risk and high-risk individuals. High-risk patients with multiple risk factors and three-vessel disease (Fig. 2B) exhibit higher systolic BP, PP, augmentation pressure, and AIx and lower diastolic BP due to increased aortic stiffness compared to low-to-intermediate-risk patients (Fig. 2A).

#### The impact of arterial stiffness on the prognosis of hypertensive patients

**Fig. 3:**
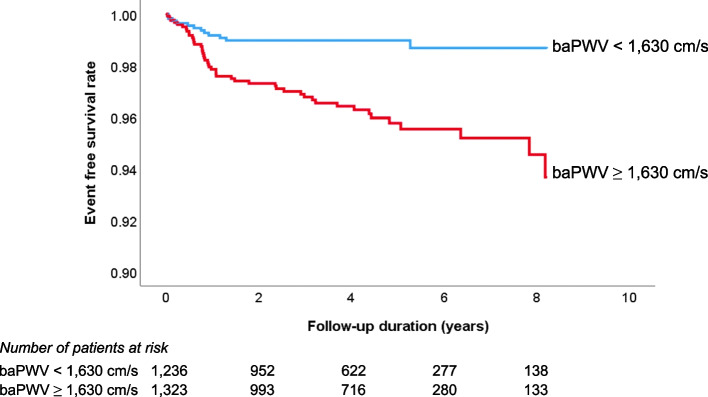
Prognostic value of baPWV in the prediction of cardiovascular events in hypertensive patients [16]. baPWV, brachial-ankle pulse wave velocity

As outlined in the previous sections, arterial stiffness plays a crucial role in causing organ damage in hypertensive patients. This organ damage is intrinsically linked to the occurrence of cardiovascular events [53]. Therefore, it’s not surprising that arterial stiffness serves as a valuable predictor for cardiovascular events in patients with hypertension. Evidence from various studies affirms this relationship. Many studies have reported that an increase in arterial stiffness leads to a deterioration in the cardiovascular prognosis of hypertensive patients [16, 58, 59]. This evidence suggests that arterial stiffness is not just a by-product but an active contributor to the worsening of cardiovascular health in hypertensive patients. Recently, our research team conducted a study involving 2,561 Korean hypertensive subjects. The results of this study demonstrated that a higher baseline baPWV was independently associated with a higher risk of cardiovascular disease occurrence. This correlation was maintained even after accounting for potential confounding factors in the multivariable analysis, highlighting the robustness of this relationship. Furthermore, in this study, the authors proposed a baPWV value of 1,630 cm/s as a cut-off for predicting cardiovascular events [16] (Fig. 3). These studies highlight the importance of monitoring arterial stiffness in hypertensive patients. It is an important risk factor for cardiovascular events, contributes to the prognosis of these patients, and provides a potential target for therapeutic intervention.

#### Therapeutic strategies targeting arterial stiffness in hypertensive patients

There are several benefits of reducing arterial stiffness in hypertensive patients. Arterial stiffness is a known independent risk factor for cardiovascular diseases [8, 9]. As such, its reduction can lead to a decreased risk of these diseases, including heart attack, stroke, and heart failure. Stiff arteries can contribute to high systolic BP, especially in older adults [7]. By lowering arterial stiffness, it may be possible to achieve better control of BP, a key component in managing hypertension and reducing related complications. Both hypertension and arterial stiffness can result in damage to important organs such as the heart, brain, and kidneys [3, 39]. By reducing arterial stiffness, the pressure load on these organs can be lessened, potentially preventing or slowing the progression of organ damage. Additionally, a reduction in arterial stiffness can improve blood flow throughout the body, leading to improved overall function and possibly reducing symptoms related to inadequate blood flow, such as fatigue or shortness of breath [60, 61].

#### How to reduce arterial stiffness in hypertensive patients?

Although not exclusive to hypertensive patients, there are several methods available to reduce arterial stiffness [5]. The cornerstone of these interventions is lifestyle modification. A balanced and healthy diet can contribute substantially towards reducing arterial stiffness. Consuming a diet rich in fresh fruits, vegetables, lean proteins, whole grains, and low-fat dairy products, while keeping sodium intake in check, is essential. These principles form the foundation of the Dietary Approaches to Stop Hypertension (DASH) diet, which is a scientifically validated method for controlling hypertension and enhancing cardiovascular health [62, 63]. Consuming a diet rich in fresh fruits, vegetables, lean proteins, whole grains, and low-fat dairy products provides essential anti-oxidants and anti-inflammatory compounds [64]. These reduce oxidative stress and inflammation, both of which contribute to arterial stiffness by damaging the endothelial lining and promoting the formation of plaques. Reducing sodium intake and adopting diets like the DASH diet can help lower BP [62, 63], which in turn reduces the mechanical strain on arterial walls. Omega-3 fatty acids, prevalent in fish, have anti-dyslipidemic properties [65]. Dyslipidemia can lead to atherosclerotic plaque buildup, further enhancing arterial stiffness. Physical exercise has also shown efficacy in reducing arterial stiffness. It appears that the consistency and regularity of exercise are more critical in lowering arterial stiffness than the specific type or intensity of the workout [66]. Meta-analysis demonstrated that exercise interventions based on aerobic, combined or isometric exercise significantly reduced PWV value in hypertensive subjects [67]. Regular physical activity improves endothelial function, promotes the release of nitric oxide, and stimulates antioxidant defense mechanisms, helping arteries remain flexible [68]. Weight management is another significant factor. By maintaining a healthy weight, individuals can stave off numerous health complications, including increased arterial stiffness and high BP [69]. However, it is important to keep in mind that the association between body mass index and arterial stiffness is not linear [70]. Moreover, proactive approaches to quitting smoking is vital, as these habits can worsen arterial stiffness and result in other deleterious health effects [71, 72]. Alcohol’s impact on arterial stiffness varies depending on the quantity consumed, and large amounts or binge drinking are known to exacerbate arterial stiffness [73]. Stress management, an often-overlooked aspect of overall health, also plays a significant role. Chronic stress is a known contributor to hypertension and arterial stiffness [74], and implementing stress management strategies like meditation, deep-breathing exercises, yoga, and ensuring adequate sleep, is vital for arterial health [75].

Pharmacological interventions can be also applied for reducing arterial stiffness. Drugs with established cardiovascular protective effects, such as RAAS blockers [76] and statins [77], have been demonstrated to be effective in this regard. Continuous positive airway pressure therapy has also shown efficacy in reducing arterial stiffness among patients with obstructive sleep apnea [78]. Despite these advancements, the current body of research on the reduction of arterial stiffness predominantly comprises small-scale studies. There is a definitive need for large-scale, comprehensive studies in the future to further validate these methods and possibly uncover more effective strategies for reducing arterial stiffness.

#### The importance and challenges of early detection of aortic stiffness

Based on what has already been described above, it is important to obtain early information on arterial stiffness in hypertensive patients. Increased arterial stiffness is a strong independent predictor of future cardiovascular events and all-cause mortality. It can help identify high-risk individuals who may require more aggressive treatment and monitoring. The detection of arterial stiffness can be useful for the early initiation of preventive and therapeutic strategies to limit the progression of vascular damage. This includes lifestyle modifications, such as dietary changes, increased physical activity, smoking cessation, and BP control, as well as the appropriate use of medication. Changes in arterial stiffness can also serve as a measure of the effectiveness of therapeutic interventions. If a particular treatment is effective in reducing arterial stiffness, it can suggest that the treatment is beneficial for vascular health and potentially reducing future cardiovascular risk. Hypertension and arterial stiffness often coexist and can exacerbate each other. Understanding the degree of arterial stiffness can provide insights into the underlying pathophysiology of an individual’s hypertension, potentially guiding more personalized treatment approaches. Studies have shown that arterial stiffness, measured by techniques like PWV, provides prognostic information beyond traditional risk factors. Patients with high arterial stiffness often have worse long-term cardiovascular outcomes, and knowing this can help shape the management plan. When patients understand that they have an increased arterial stiffness that can be improved with healthy lifestyle changes, it may provide additional motivation for them to make and maintain these changes. In summary, early detection of arterial stiffness in hypertensive patients can guide therapeutic decisions, help monitor the effectiveness of interventions, improve risk stratification, and potentially improve outcomes.

## Conclusions

Hypertension and arterial stiffness are closely related to each other. When BP is high, arterial stiffness progresses, and conversely, when arterial stiffness progresses, BP also increases. Arterial stiffness information can be used in hypertensive patients to predict the cardiovascular risk. Several methods are available for measuring arterial stiffness; however, the use of non-invasive techniques makes the information highly beneficial in treating hypertensive patients. The suggestion has been made that a healthy lifestyle, RAAS blockers, and statins are beneficial in reducing arterial stiffness. Further research is required to determine if a treatment strategy that ameliorates arterial stiffness can enhance the prognosis of patients with hypertension.

## Data Availability

Not applicable.

## Abbreviations

AIx: Augmentation index
baPWV: Brachial-ankle pulse wave velocity
BP: Blood pressure
cfPWV: Carotid-femoral pulse wave velocity
LVH: Left ventricular hypertrophy
MMP: Matrix metalloproteinases
NO: Nitric oxide
PP: Pulse pressure
PWV: Pulse wave velocity
RAAS: Renin–angiotensin–aldosterone system
